# Whole exome sequencing of benign pulmonary metastasizing leiomyoma reveals mutation in the *BMP8B* gene

**DOI:** 10.1186/s12881-018-0537-5

**Published:** 2018-01-31

**Authors:** Deniss Sõritsa, Hindrek Teder, Retlav Roosipuu, Hannes Tamm, Triin Laisk-Podar, Pille Soplepmann, Alan Altraja, Andres Salumets, Maire Peters

**Affiliations:** 1 Institute of Clinical Medicine, Department of Obstetrics and Gynaecology, University of Tartu, Tartu, Estonia; 2 Elite Clinic, Sangla 63, 50407 Tartu, Estonia; 3 Competence Centre on Health Technologies, Tartu, Estonia; 4 Institute of Biomedicine and Translational Medicine, Department of Biomedicine, University of Tartu, Tartu, Estonia; 5 Department of Pathology, Tartu University Hospital, Tartu, Estonia; 6 Tartu University Hospital’s Women’s Clinic, Tartu, Estonia; 7 Department of Pulmonary Medicine, University of Tartu, Tartu, Estonia; 8 Lung Clinic, Tartu University Hospital, Tartu, Estonia; 9 Department of Obstetrics and Gynaecology, University of Helsinki and Helsinki University Hospital, Helsinki, Finland

**Keywords:** Benign metastasizing leiomyoma, BMP8B, Endometriosis, GnRH agonists, Pulmonary lesion, Somatic gene mutation

## Abstract

**Background:**

Benign metastasizing leiomyoma (BML) is an orphan neoplasm commonly characterized by pulmonary metastases consisting of smooth muscle cells. Patients with BML have usually a current or previous uterine leiomyoma, which is therefore suggested to be the most probable source of this tumour. The purpose of this case report was to determine the possible genetic grounds for pulmonary BML.

**Case presentation:**

We present a case report in an asymptomatic 44-year-old female patient, who has developed uterine leiomyoma with subsequent pulmonary BML. Whole exome sequencing (WES) was used to detect somatic mutations in BML lesion. Somatic single nucleotide mutations were identified by comparing the WES data between the pulmonary metastasis and blood sample of the same BML patient. One heterozygous somatic mutation was selected for validation by Sanger sequencing. Clonality of the pulmonary metastasis and uterine leiomyoma was assessed by X-chromosome inactivation assay.

**Conclusions:**

We describe a potentially deleterious somatic heterozygous mutation in bone morphogenetic protein 8B (*BMP8B*) gene (c.1139A > G, Tyr380Cys) that was identified in the pulmonary metastasis and was absent from blood and uterine leiomyoma, and may play a facilitating role in the metastasizing of BML. The clonality assay confirmed a skewed pattern of X-chromosome inactivation, suggesting monoclonal origin of the pulmonary metastases.

**Electronic supplementary material:**

The online version of this article (10.1186/s12881-018-0537-5) contains supplementary material, which is available to authorized users.

## Background

Benign metastasizing leiomyoma (BML) is a rare condition that occurs mainly in premenopausal women [1] and is characterized most commonly by pulmonary and lymph node metastases consisting of mature smooth muscle cells [2]. BML is usually associated with a current or previous uterine leiomyoma, and based on histological findings and positive staining for oestrogen and progesterone receptors [3], uterine leiomyomas have been proposed as the most probable source of this disease. The lesions are slow-growing and usually the disease has a favourable prognosis. However, there are no standardized guideline-based treatments for BML and surgical removal along with hormone therapy is the most frequently used management [3, 4]. Although uterine leiomyomas are the most probable source of the metastases, the pathogenesis of BML has still remained enigmatic. Among other theories, it has been suggested that BML may emerge from lymphatic and hematological spread of the uterine tissue or via coelomic metaplasia [5]. The origin of metastases from uterine tissues is supported by molecular studies demonstrating the same clonal origin of the metastases of BML and original uterine leiomyomas [6–8]. Almost half of the uterine leiomyomas are cytogenetically abnormal and furthermore, they possess gene mutations, with mediator complex subunit 12 as the most frequently affected gene [9]. The occurrence of chromosomal aberrations in BML metastases has also been shown [10, 11]; however, only one study has explored single nucleotide variations (SNVs) and short insertions/deletions (indels) in metastases using massively parallel sequencing of a panel of 409 cancer-related genes [8]. Here, we present the results of whole exome sequencing (WES) of the BML metastasis from a 44-year-old female patient, who had developed uterine leiomyoma with subsequent pulmonary BML.

## Case presentation

**Fig. 1:**
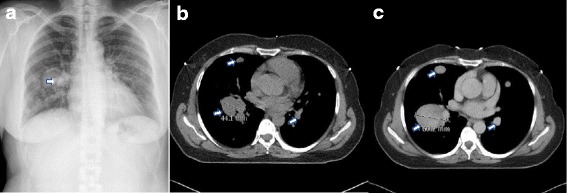
X-ray (**a**) and high resolution computed tomography (**b**, **c**) images of the BML patient from different examinations (November 2013, January 2015 and June 2015, respectively). Pulmonary metastases are shown by arrows

**Fig. 2:**
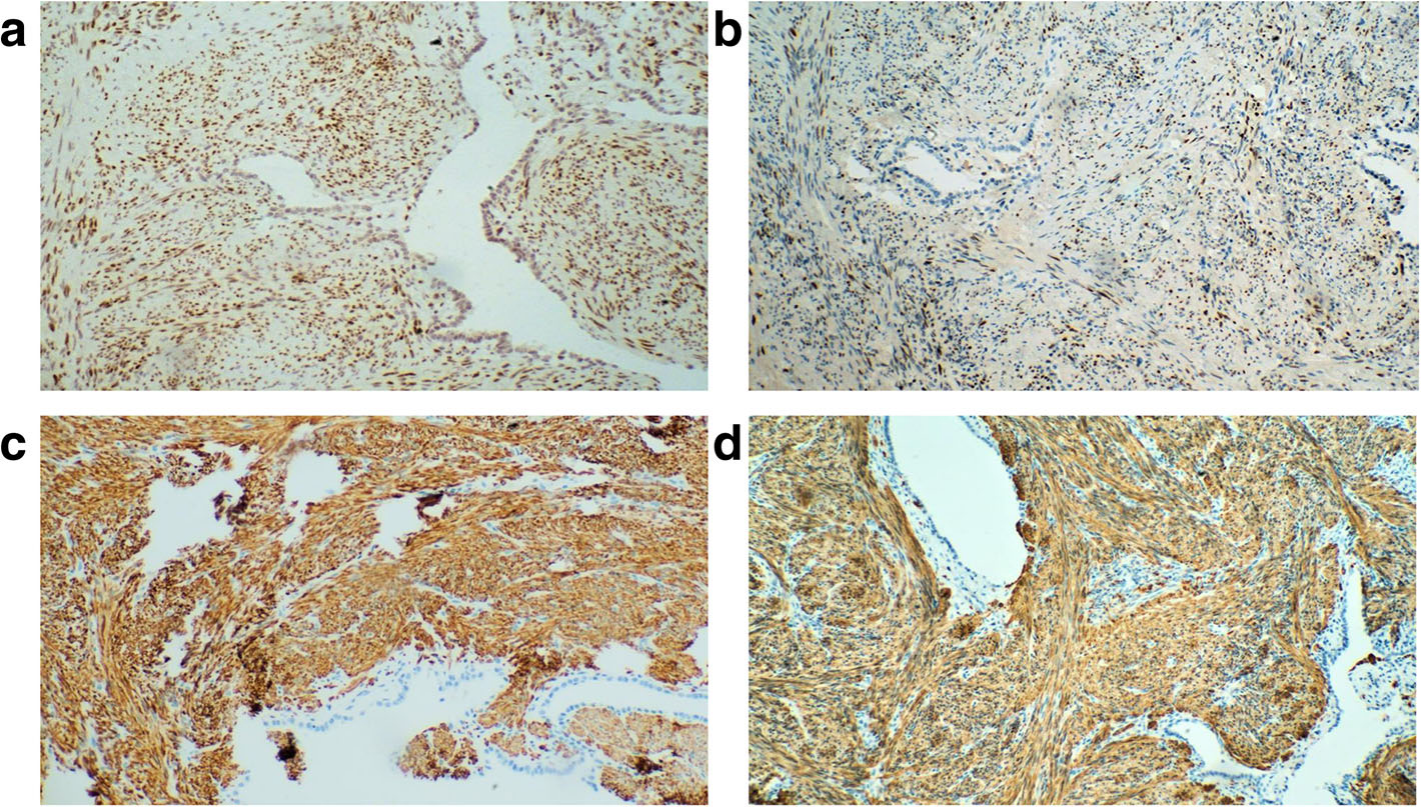
Immunohistochemistry of the BML patient’s lung metastasis showing positivity for oestrogen receptor α (**a**), progesterone receptor (**b**), desmin (**c**) and α-smooth muscle actin (d). Original magnifications × 100

**Fig. 3:**
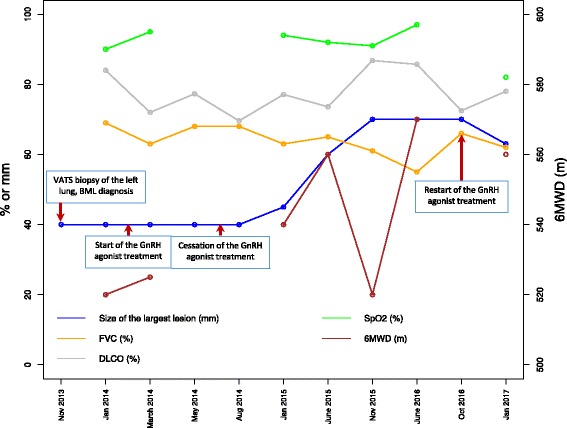
The dynamics of pulmonary function parameters and the size of the largest lung metastasis of the BML patient. The size of the largest lung metastasis is given in mm’s (left side Y-axis), diffusing capacity of the lungs for carbon monoxide (DLCO) and post-bronchodilator forced vital capacity (FVC) are given as % predicted (left side Y-axis), the six-minute walk distances (6MWD) are given in meters (right side Y-axis), and the lowest oxygen saturation (SpO_2_) during the six-minute walk test is shown as % (left side Y-axis) of the lower limit of normal. VATS - video-assisted thoracoscopic surgery

Caucasian female, at the age of 34, underwent abdominal uterine myomectomy because of anaemia, but not all nodules were removed. Six years later, she underwent laparoscopy-assisted supracervical hysterectomy (the weight of the uterus was 288 g) because of recurrent anaemia and uterine leiomyomas. Pathohistologically, the specimens presented as a uterine smooth muscle tumour without evidence of malignancy. Three years later, at the age of 43, laparoscopic salpingo-oophorectomy was performed because of a 6-cm endometrioma in the right ovary, and superficial endometriotic lesions on the left ovary were coagulated. In the same year, during a routine chest X-ray examination, multiple well-defined round pulmonary nodules of various sizes up to 40 mm in diameter were detected (Fig. 1a). After 1-year follow-up, a high-resolution computed tomography (HRCT)-scan of the chest confirmed the largest nodule of having a diameter of 40 mm. Video-assisted thoracoscopic surgery of the left lung with a biopsy was performed in November 2013. Immunohistochemistry (IHC) analysis of lung lesion specimens revealed positive staining for oestrogen receptor α, progesterone receptor, desmin and α-smooth muscle actin (Fig. 2a-d), while staining for Ki67 revealed low mitotic activity (< 1–5%). On the basis of pathohistology and IHC signature, the diagnosis of BML was established, and subsequently, the patient received a treatment with gonadotropin-releasing hormone (GnRH) agonist, goserelin (Zoladex, AstraZeneca UK Ltd. London, UK), for 6 months. The 4-month and 6-month follow-up chest X-ray showed that lesions, formerly undergoing progressive enlargement, had remained unchanged (Fig. 3). However, the treatment was discontinued because of the disagreeable side effects (weight gain of > 10 kg, flushing and profuse sweating), and dietary and lifestyle modifications to address the overweight issue were suggested. After a 5-month treatment-free period (in January 2015), a new chest X-ray examination revealed enlargement of the metastases (the size of the largest nodule has increased by 4 mm reaching 44 mm, Fig. 1b) and the patient complained of shortness of breath occurring more easily. Six months later, a new HRCT-scan revealed further enlargement of the metastases as the diameter of the largest lesion was 60 mm (Figs. 1c and 3). The patient still decided to postpone the next treatment because of the fear of the side-effects. After the following 5 months (November 2015), additional enlargement of metastases was detected, with the largest lesion gaining a diameter of 70 mm. The monitoring continued without treatment as the patient reported her general health and wellbeing to be satisfactory (presumably because of the normalization of body weight), and refused from GnRH agonist treatment. Seven months later, the follow-up HRCT-scan showed the majority of the metastases had not changed. In October 2016, the second 6-month treatment with goserelin was initiated because of the further enlargement of some lesions, and the last chest X-ray in January 2017 showed stabilization of the size of majority of the lesions; moreover, the largest nodules had decreased by about 10% in size. The patient had no complaints except for weight regain.

During the visits to the pulmonologist, several functional tests were performed (Fig. 3). The values for diffusing capacity of the lungs for carbon monoxide (DLCO) were either within the normal range or slightly below the lower limit of normal (normal defined as 74% of the predicted value [12]), being in the range of 69.6–86.8% predicted. Post-bronchodilator forced vital capacity (FVC) values were below the lower limit of normal (defined as 80% of the predicted value for the patient), being 55.0–69.0% predicted. Patient’s 6-min walk test (6MWT) results were within the normal range, with absolute values of the 6-min walk distance (6MWD) being 520–570 m (100–139% of the predicted lower limit of normal values), accompanied with mild breathlessness during the 6MWT, but following by an adequate recovery. The values of oxygen saturation measured by pulse oximetry (SpO_2_) during the 6MWT were 90–97%, i.e. normal or slightly below the lower limit of normal (94%), with only one exceptional registered value indicative of desaturation (81%).

To find possible genetic causes of BML, WES of blood and pulmonary metastasis was performed (detailed description of the WES is given in Additional file 1).

**Fig. 4:**
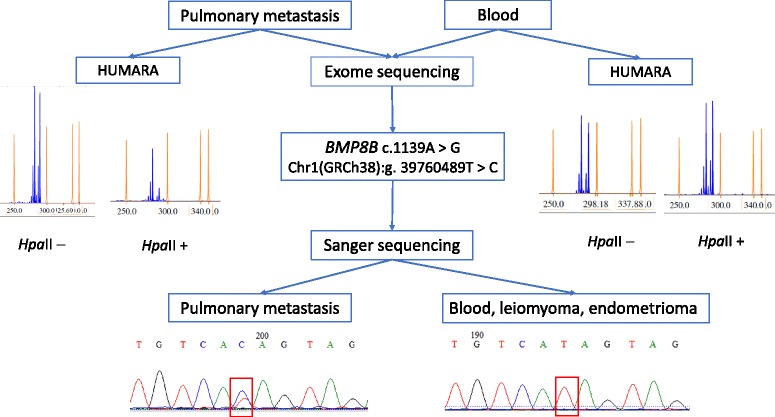
Validation of mutation in the *BMP8B* gene by Sanger sequencing and X-chromosome inactivation assay (HUMARA) of pulmonary metastasis and peripheral blood of the BML patient. *Hpa*II+ denotes enzyme-digested DNA and *Hpa*II− means undigested DNA. Pulmonary metastasis demonstrates a pattern of nonrandom X-chromosome inactivation with a nearly complete absence of the androgen receptor longer allele after *Hpa*II digestion. The DNA sequence electropherogram shows the reverse strand of the *BMP8B* gene

The mean sequencing depth was 57× for both DNA samples. The manual inspection of the metastasis and peripheral blood sample data did not reveal regions with aberrant sequencing coverage, suggesting that no large chromosomal alterations (deletions or duplications) were present in the coding areas. After filtering the exome data to exclude germline variants, a total of 121 potential somatic mutations (SNVs and short indels) affecting 109 genes (Additional file 2) were identified from the protein-coding gene regions of DNA from the pulmonary metastasis. The discovered somatic variants were manually inspected and the candidate mutations were filtered according to the following criteria: (1) mutations in splice regions, and coding exons that affect amino acids; (2) predicted deleterious by SIFT or PolyPhen2; (3) as the pulmonary metastasis was histologically estimated to consist almost entirely of smooth muscle cells (> 90%) then in case of heterozygous mutations, similar allelic depths for the reference and alternative allele were expected; and (4) reported or suggested to be involved in tumorigenesis. After rigorous filtering, one heterozygous mutation in the *BMP8B* (bone morphogenetic protein 8B) gene was found to correspond to the filtering criteria. The sequencing depths of the alternative and reference alleles for BML and germline/blood DNA were 57/60 and 0/97, respectively. PolyPhen 2 and SIFT predicted this variant as probably damaging (score 0.994) and deleterious, respectively. Sanger sequencing confirmed the presence of an amino acid-affecting heterozygous mutation (c.1139A > G, Y380C) in the seventh exon of the *BMP8B* gene in the pulmonary metastasis (Fig. 4). The tissue specimens of endometrioma and leiomyoma from the same patient were also examined for this variation, but neither of the tissues had this mutation.

The human androgen receptor (AR) based X-chromosome inactivation assay (HUMARA) was used (as described elsewhere [13]) to determine the clonality status of the pulmonary metastasis and the uterine leiomyoma. Analysis of both of these specimens revealed a similar pattern of skewed X-chromosome inactivation, with an inactivation of the chromosome carrying the shorter AR allele (Fig. 4 and Additional file 3). On the contrary, the blood DNA demonstrated a random inactivation of X-chromosomes.

## Discussion and conclusions

We describe WES results of the patient, who has developed uterine leiomyoma with subsequent pulmonary BML. In addition, the patient suffered from endometriosis presenting as a large endometrioma of the right ovary and superficial lesions on the left ovary. The mechanisms used to explain the pathogenesis of endometriosis [14, 15] can also apply to BML. Hence, it has been proposed that BML may evolve from lymphatic and hematological spread, coelomic metaplasia and intraperitoneal seeding from ruptured leiomyoma [16]. Furthermore, there is an evidence from molecular studies supporting the hypothesis that leiomyoma could be the source of this benign tumour, as X-chromosome inactivation assay has confirmed the clonal origin of uterine tumours and BML [6, 7, 17]. A very recent study that showed shared SNVs between synchronous pulmonary and uterine leiomyomata gives further strong evidence to support a clonal relationship between these two tumours [8]. Copy number variance (CNV) analysis has also been used to explore the origin of metastases and in some cases, similar chromosomal aberrations have been found in tumours of both locations, confirming their genetic relationship [10, 18]. In addition, the presence of consistent chromosomal deletions of 19q and 22q in all five investigated BML cases was demonstrated by Nucci et al. [11]. However, these deletions are not characteristic to rearrangements frequently found in uterine leiomyomas, with chromosomes 6, 7, 10 and 12 most commonly affected [19], and the authors proposed that BML may arise from a biologically distinct minority of uterine leiomyomas with an innate metastatic potential [11]. However, in some cases, CNV analysis showed balanced karyotype of BML specimens without any changes in DNA copy numbers [6, 17], as was also the case for the patient described in the current study.

Most of BML cases have been detected on routine chest X-rays. BML is a rare condition that should always be considered in case of incidental lung findings in women with a previous or coincident history of uterine leiomyoma. The presence of oestrogen and progesterone receptors supports a connection between BML and female reproductive tract and makes the basis for the rationale for using hormonal treatment [5, 20]. GnRH agonists are often used and are described to give favourable therapeutic outcomes in terms of preventing the enlargement of nodules [21]. According to the published data, the periods of GnRH agonist treatment vary from 3 to 42 months [22, 23]. In the current case, we had a unique opportunity to longitudinally observe the growth dynamics of the pulmonary metastases, which followed the pattern of goserelin treatment. The enlargement of metastases, when patient discontinued the treatment because of the unfavourable side effects, provided the rationale for continuing the hormone therapy.

According to our knowledge, this is the first study using WES to explore SNVs of BML specimen. Even though WES revealed several somatic candidate-mutations in the pulmonary BML tissue, only a heterozygous mutation in the *BMP8B* gene corresponded to the criteria we used to select the probable disease-associated mutations. Wu et al. used the Ion AmpliSeq Comprehensive Cancer Panel that targets the exons of 409 tumor suppressor genes and oncogenes to find possible mutations in three cases of synchronous pulmonary and uterine leiomyomata tissues [8]. Somatic mutations were found in all investigated tissues, and shared alterations between uterine leiomyoma and pulmonary metastases were found in two out of three cases, but no recurrent mutations were observed. The authors concluded that the probability of finding the same alterations in different cases was very low as none of the detected mutations has commonly been described in leiomyomas. Unfortunately, as the *BMP8B* gene is not a common cancer-gene, it is also not included in the Cancer Panel and therefore the presence or absence of mutations in this gene could not be evaluated in the study by Wu and colleagues. BMPs represent a family of signalling molecules that belong to the transforming growth factor-β superfamily of proteins and play crucial roles in all organ systems [24]. In cancer, BMPs can either suppress or promote tumorigenesis and are involved in the metastasis of cancer cells [25]. *BMP8B* is a gene encoding a protein with a role in spermatogenesis [26] and in thermogenesis [27]. *BMP8B* has not been detected as a mutational cancer driver; however, there are some reports describing altered expression of the *BMP8B* gene in cancers [28–30]. Reduced *BMP8B* mRNA expression has been detected in tumour tissues compared to adjacent normal tissues, whereas experimental induction of the *BMP8B* overexpression inhibited cell growth [29], proposing that *BMP8B* could act as a tumour suppressor gene [28]. This gene locates on the short arm of chromosome 1 and the mutation detected in pulmonary metastasis locates in the last (seventh) exon of the gene. The mutation affects amino acid constitution of the protein (tyrosine-to-cysteine substitution) and because both PolyPhen2 and SIFT annotate it as probably damaging, we can speculate that it plays a role in BML lesions’ growth and metastasis. Unfortunately, we had no possibility to test the impact of the mutation on protein function level as we had only limited FFPE material of pulmonary metastasis. The same mutation was not present in the leiomyoma tissue of the patient; and therefore, we can assume that a) the mutation has occurred in the lung BML lesion only; b) the patient suffered from multiple leiomyomas, and the pulmonary BML did not arise from that particular uterine leiomyoma tissue analysed in the current study; or c) BML does not originate from leiomyoma. It has been demonstrated that leiomyomas develop as clonal lesions but multiple nodules in a single uterus may have different clonal origin [31] and harbour different chromosomal aberrations [32]. Also, the study by Wu et al. revealed shared somatic mutations only in two out of three pairs of uterine leiomyoma and BML metastases [8]. Our clonality analysis confirmed the non-random X-chromosome inactivation in both uterine leiomyoma and pulmonary metastasis, with the same allele being inactivated. However, this finding can also be coincidental showing just the clonal origin of these tumours.

In addition to leiomyoma, the patient suffered from endometriosis that resulted in the development of a large ovarian endometrioma. According to the theories of endometriosis development, endometrial tissue is the most probable source of endometriosis. Therefore, we examined the endometrioma specimen for the presence of the *BMP8B* gene mutation, but the analysis revealed only a wild-type allele. Thus, the co-occurrence of endometriosis and BML in the current patient is probably random, as previously only a few reports have described BML-associated endometriosis [33, 34].

In conclusion, we described the longitudinal follow-up of a patient suffering from BML. We demonstrated that GnRH agonist therapy gave favourable outcome and treatment discontinuation caused enlargement of the lung lesions. Genetic analysis revealed a *BMP8B* gene mutation in the lung lesion; however, the link between mutations in the *BMP8B* gene and pulmonary BML needs to be corroborated by further studies involving additional patients and exploring the functional consequences of the here-described mutation.

## Additional files

Additional file 1: Supplementary methods. DNA extraction and sequencing. (DOCX 21 kb)

Additional file 2: Table S1. Summary of somatic variants identified in pulmonary metastasis of the BML patient by whole exome sequencing. (XLSX 34 kb)

Additional file 3: Figure S1. A non-random X-chromosome inactivation pattern of leiomyoma specimen of the BML patient. *Hpa*II + denotes enzyme-digested DNA and *Hpa*II − means undigested DNA. (PPTX 46 kb)

## Abbreviations

BML: Benign metastasizing leiomyoma
BMP8B: Bone morphogenetic protein 8B
CNV: Copy number variance
FFPE: Formalin-fixed, paraffin-embedded
GnRH: Gonadotropin-releasing hormone
HRCT: High-resolution computed tomography
IHC: Immunohistochemistry
SNV: Single nucleotide variation
WES: Whole exome sequencing
