# Supplementary material for: Whole exome sequencing of benign pulmonary metastasizing leiomyoma reveals mutation in the BMP8B gene
Source: BMC Med Genet. 2018 Jan 31;19:20. doi: 10.1186/s12881-018-0537-5 (PMC5793349; doi:10.1186/s12881-018-0537-5)
Supplement: Supplementary file 1 — Supplementary methods. DNA extraction and sequencing. (DOCX 21 kb) [file 12881_2018_537_MOESM1_ESM.docx]

***DNA extraction and sequencing***

Tissue specimens of uterine leiomyoma, endometrioma and a pulmonary metastasis from a single patient were collected during surgeries at Tartu University Hospital (Tartu, Estonia). The tissue specimens were fixed in 10 % neutral buffered formalin, dehydrated, embedded in paraffin and evaluated by the pathologist. Immunohistochemistry (IHC) analyses were performed at Tartu University Hospital’s Pathology Service. The pulmonary metastasis was histologically estimated to consist mostly of smooth muscle cells (>90%). For DNA extraction, two to four 5 μm sections from archived formalin-fixed, paraffin-embedded (FFPE) tissue blocks were collected in 1.5 mL microcentrifuge tubes and DNA was extracted using QIAamp DNA FFPE Tissue Kit (Qiagen, Hilden, Germany). Blood DNA was extracted using QIAamp Blood Mini Kit (Qiagen) according to the manufacturer’s protocols.

For WES of blood and pulmonary metastasis DNA, the library construction and sequencing were performed by Centogene AG (Rostock, Germany). Whole exome libraries were prepared in accordance with Nextera Rapid Capture Exome Enrichment protocol and sequenced using Illumina HiSeq system (Illumina, USA). The quality control of raw sequencing data was performed using FastQC version 0.11.5 [[1](#_ENREF_1)]. The paired-end reads were mapped to the human reference genome GRCh38 using BWA-MEM algorithm in bwakit version 0.7.15 [[2](#_ENREF_2)]. Duplicated reads were identified using Picard version 2.8.0 [[3](#_ENREF_3)]. Base quality score recalibration of the sequencing data was carried out using Genome Analysis Toolkit (GATK) version 3.7 [[4](#_ENREF_4)]. The known polymorphic variants from dbSNP build 146, and from Mills and 1000G gold standard indel set were masked before variant discovery.

Somatic single nucleotide variants and indels were identified by comparing the pulmonary metastasis and blood sample using GATK MuTect2. According to the GATK Best Practice recommendations, COSMIC version 79 and dbSNP databases were taken into account in variant discovery [[5](#_ENREF_5)]. The discovered somatic variants were manually inspected and the candidate mutations were filtered according to the following criteria: (1) mutations in splice regions, and coding exons that affect amino acids; (2) predicted deleterious by SIFT or PolyPhen2; (3) in case of heterozygous mutations, similar allelic depths for the reference and alternative allele are present; and (4) reported or suggested to be involved in tumorigenesis.

One heterozygous somatic mutation was selected for validation by Sanger sequencing. Primers specific to the region of interest were designed using Primer3 software [[6](#_ENREF_6)] and sequencing was performed on both DNA strands. Primer sequences and PCR conditions are available on request.

**References**

1. A quality control tool for high throughput sequence data. https://wwwbioinformaticsbabrahamacuk/projects/fastqc/. Accessed 16 January 2017.

2. Li H. Aligning sequence reads, clone sequences and assembly contigs with BWA-MEM. arXiv:13033997v2 [q-bioGN]. 2013

3. Picard. http://broadinstitutegithubio/picard. Accessed 16 January 2017.

4. McKenna A, Hanna M, Banks E, Sivachenko A, Cibulskis K, Kernytsky A, et al. The Genome Analysis Toolkit: a MapReduce framework for analyzing next-generation DNA sequencing data. Genome Res. 2010;20(9):1297-303.

5. Van der Auwera GA, Carneiro MO, Hartl C, Poplin R, Del Angel G, Levy-Moonshine A, et al. From FastQ data to high confidence variant calls: the Genome Analysis Toolkit best practices pipeline. Curr Protoc Bioinformatics. 2013;43:11.10.1-33.

6. Untergasser A, Cutcutache I, Koressaar T, Ye J, Faircloth BC, Remm M, et al. Primer3--new capabilities and interfaces. Nucleic Acids Res. 2012;40(15):e115.
