# Supplementary figures and images for: Whole exome sequencing of benign pulmonary metastasizing leiomyoma reveals mutation in the BMP8B gene
Source: BMC Med Genet. 2018 Jan 31;19:20. doi: 10.1186/s12881-018-0537-5 (PMC5793349; doi:10.1186/s12881-018-0537-5)

## Slide 1
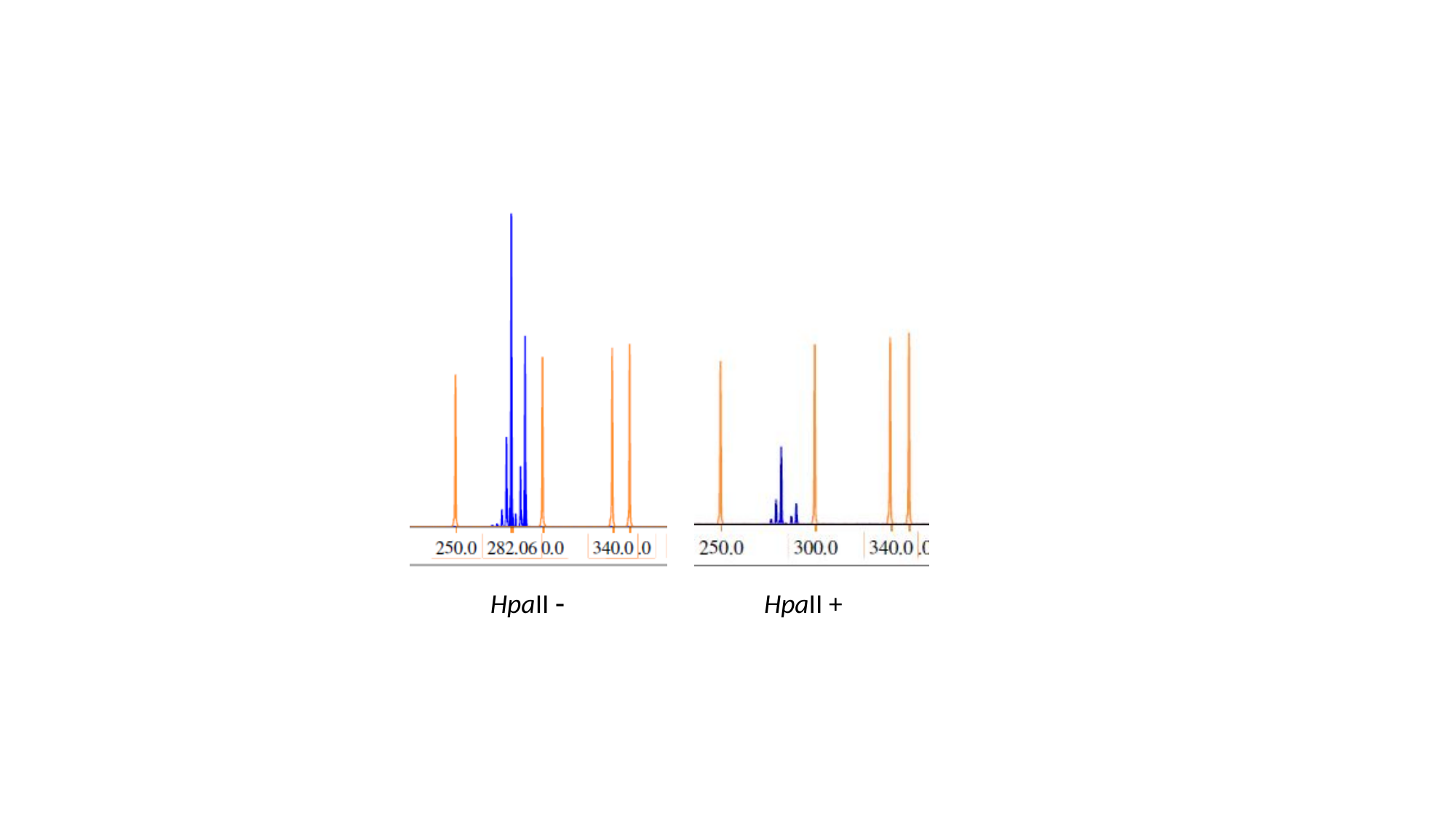

HpaII −
HpaII +

Supplement: Supplementary file 3 — A non-random X-chromosome inactivation pattern of leiomyoma specimen of the BML patient. HpaII + denotes enzyme-digested DNA and HpaII − means undigested DNA. (PPTX 46 kb) [file 12881_2018_537_MOESM3_ESM.pptx]
